# Human Cardiac Progenitor Cells Enhance Exosome Release and Promote Angiogenesis Under Physoxia

**DOI:** 10.3389/fcell.2020.00130

**Published:** 2020-03-06

**Authors:** Julie A. Dougherty, Nil Patel, Naresh Kumar, Shubha Gururaja Rao, Mark G. Angelos, Harpreet Singh, Chuanxi Cai, Mahmood Khan

**Affiliations:** ^1^Department of Emergency Medicine, The Ohio State University Wexner Medical Center, Columbus, OH, United States; ^2^Dorothy M. Davis Heart Lung and Research Institute, The Ohio State University Wexner Medical Center, Columbus, OH, United States; ^3^Department of Physiology and Cell Biology, The Ohio State University Wexner Medical Center, Columbus, OH, United States; ^4^Division of Cardiac Surgery, Department of Surgery, The Ohio State University Wexner Medical Center, Columbus, OH, United States

**Keywords:** cardiac progenitor cells, stem cells, hypoxia, extracellular vesicles, angiogenesis, cardiac repair

## Abstract

Studies on cardiac progenitor cells (CPCs) and their derived exosomes therapeutic potential have demonstrated only modest improvements in cardiac function. Therefore, there is an unmet need to improve the therapeutic efficacy of CPCs and their exosomes to attain clinically relevant improvement in cardiac function. The hypothesis of this project is to assess the therapeutic potential of exosomes derived from human CPCs (hCPCs) cultured under normoxia (21% O_2_), physoxia (5% O_2_) and hypoxia (1% O_2_) conditions. hCPCs were characterized by immunostaining of CPC-specific markers (NKX-2.5, GATA-4, and c-kit). Cell proliferation and cell death assay was not altered under physoxia. A gene expression qPCR array (84 genes) was performed to assess the modulation of hypoxic genes under three different oxygen conditions as mentioned above. Our results demonstrated that very few hypoxia-related genes were modulated under physoxia (5 genes upregulated, 4 genes down regulated). However, several genes were modulated under hypoxia (23 genes upregulated, 9 genes downregulated). Furthermore, nanoparticle tracking analysis of the exosomes isolated from hCPCs under physoxia had a 1.6-fold increase in exosome yield when compared to normoxia and hypoxia conditions. Furthermore, tube formation assay for angiogenesis indicated that exosomes derived from hCPCs cultured under physoxia significantly increased tube formation as compared to no-exosome control, 21% O_2_, and 1% O_2_ groups. Overall, our study demonstrated the therapeutic potential of physoxic oxygen microenvironment cultured hCPCs and their derived exosomes for myocardial repair.

## Introduction

Myocardial infarction (MI) is responsible for the death of one American every 40 s (Benjamin et al., 2017). MI is caused by a limited supply of blood and oxygen to the heart, which leads to cardiac dysfunction, fibrosis, and, ultimately, heart failure. Post-MI, there is a permanent loss of cardiomyocytes and scar tissue formation that results in irreversible damage and maladaptation, which affects cardiac function (Prabhu and Frangogiannis, 2016). Existing therapies have been used to prevent additional damage to the heart muscle and reduce the risk of future MI, however, they only slow the progression to heart failure. Therefore, effective cardiac repair is essential to restore function of the heart following MI. Currently, cell-based therapies to aid in cardiac tissue regeneration, such as the application of stem cells, serve to be the most promising therapeutic option today (Krishna et al., 2011; Choi et al., 2015). Nevertheless, optimal cell type and conditions have not yet been identified for clinically relevant repair.

Stem cells have been previously used as a cell-based therapy to restore cardiac function. Embryonic stem cells (ESCs) hold promise as a cellular therapy because they are pluripotent; however, the clinical use of this cell line raises several ethical issues and political controversies. Today, adult stem cells are the only cells utilized as a cell-based therapy to treat MI in the United States (Le and Chong, 2016). Cardiac progenitor cells (CPCs) have been of particular interest for stem cell therapy in the treatment of MI since they have the ability to differentiate into three cardiac lineages: cardiomyocytes, smooth muscle cells, and endothelial cells. However, cell survival post-transplantation is very poor. Interestingly, despite this, moderate improvements in cardiac function are observed (den Haan et al., 2012; Noort et al., 2012; van der Spoel et al., 2012; Zuo et al., 2012; Bao et al., 2017; Wu et al., 2017), which points toward a paracrine mechanism of repair.

Extracellular vesicles, including exosomes and microparticles, have recently become of particular interest as they have been identified to be key players in paracrine signaling (Hergenreider et al., 2012; Raposo and Stoorvogel, 2013; Maas et al., 2017; Sullivan et al., 2017) and have become a major focus of research in this area. Exosomes are 50–150 nm (Yanez-Mo et al., 2015) vesicles formed by inward budding of endosomal membranes (Thery, 2011). Microvesicles are larger, 100–1000 nm (Cocucci and Meldolesi, 2015), and directly bud from the cell membrane (Colombo et al., 2014). Despite their differences, practical isolation of the two types of particles is difficult and we will refer to exosomes and microvesicles collectively as EVs. These EVs are nanoparticles that contain lipids (Record et al., 2014), proteins (Choi et al., 2015), and nucleic acids (Gezer et al., 2014; Ahadi et al., 2016; Ohno and Kuroda, 2016), which are specifically packaged depending on their source cell type and microenvironment (Xiao et al., 2016; Dougherty et al., 2017). EVs have already demonstrated therapeutic potential in treating the heart post-MI. In 2010, a study demonstrated that exosomes secreted by MSCs reduce myocardial ischemia/reperfusion injury via a mouse Langendorff heart model. In this study, exosomes were administered prior to reperfusion and results showed decreased infarct size (Lai et al., 2010). Another study demonstrated proangiogenic activities of atrial appendage CPC-derived EVs both *in vitro* and *in vivo* (Barile et al., 2014). Specifically, this study showed that these EVs inhibited cardiomyocyte apoptosis and enhanced angiogenesis, as they were enriched in miRNAs with anti-apoptotic and proangiogenic activities (Barile et al., 2014). Oxygen concentration used for culture were not reported for either study, thus, one then assumes cells were cultured at standard laboratory cell culture conditions of 21% O_2_.

The role of oxygen is severely critical in the survival of any type of cell line including stem cells. Oxygen controls the cellular microenvironment, serving as both a metabolic substrate and a signaling molecule (Abdollahi et al., 2011). Standard cell culturing protocols utilize 21% O_2_ for culturing and maintaining the cells. These conditions are considered normoxia, as it is the atmospheric level of oxygen. On the contrary, in an *in vivo* scenario, the oxygen microenvironments for cells are much lower than 21% O_2_. The relative oxygen concentration of arterial blood is approximately 12% and most tissue is around 3.4 to 6.8% with concentration varying based on location (reviewed in Abdollahi et al., 2011; McKeown, 2014). McKeown proposes that 5% O_2_ be termed “physoxia” as it is a better estimate of tissue oxygenation (McKeown, 2014). Conversely, hypoxic culture of cells affects their functional behavior and can have therapeutic applications. In two different studies, hypoxic culture (1% O_2_) of adipose stromal cells enhanced cytokine production and increased their angiogenic properties (Rehman et al., 2004; Thangarajah et al., 2009). Also, hypoxic culture (2% O_2_) of stem cells has demonstrated various benefits including a 30-fold increase in the expansion of cells compared to normoxic conditions in a study utilizing human bone marrow-derived mesenchymal stem cells (BM-MSCs) (Grayson et al., 2007). Another study demonstrated that hypoxic preconditioning (1% O_2_ hypoxia for 6 h) enhanced CPC function by demonstrating increased invasion ability and pro-survival pathway activation (Hernandez et al., 2018). Thus, culturing cells *in vitro* at physoxic and hypoxic conditions mimics the *in vivo* microenvironment and that of the ischemic heart post-MI. Additionally, previous studies have reported that short-term hypoxic culture resulted in enhanced exosome release from mouse CPCs and altered their molecular contents (Gray et al., 2015; Barile et al., 2017). Therefore, the focus of this paper was to investigate whether low-oxygen culturing (5 or 1% O_2_) of hCPCs modulates hypoxia signaling genes and their derived exosomes for cardiac repair post-MI.

## Materials and Methods

### Culture of Cardiac Progenitor Cells

Human cardiac progenitor cells (hCPCs) were isolated from the right atrial appendage and sorted for expression of c-kit cell surface marker, as described previously (Zhang et al., 2017). Cells were used at passage 7–10 for these studies. Cells were initially cultured for 48 h at normoxic conditions (37°C, 21% O_2_) then placed in medium with exosome-depleted FBS (SBI, Palo Alto, CA, United States) and continuously cultured at normoxic condition of 21% O_2_ physoxic condition of 5% O_2_ or hypoxic condition of 1% O_2_ using a controlled C-chamber incubator (ProOx P110 O_2_ Controllers, BioSperix, Parish, NY, United States). Media was refreshed every other day, retaining 20% of conditioned media. Phase-contrast images were captured using a DM IL LED microscope and MC170 HD digital camera (Leica Microsystems, Inc., Buffalo Grove, IL, United States).

### Immunofluorescent Staining

Cells were seeded on glass cover-slips coated with 0.5% gelatin and cultured at 21, 5, and 1% O_2_ for 48 h. Cells were fixed with 4% paraformaldehyde in PBS for 10 min at room temperature, permeabilized with 0.25% Triton-X-100 in PBS for 20 min at 4°C, and incubated overnight at 4°C with antibodies in antibody dilution buffer (1% w/v BSA, 0.3% Triton-X-100 in PBS): NKX-2.5 (1:25, PA5-49431, Invitrogen, Carlsbad, CA, United States), GATA-4 (1:300, PA1-102, Invitrogen, Carlsbad, CA, United States), and c-kit (1:25, MA5-12944, Invitrogen, Carlsbad, CA, United States). Cells were washed three times for 5 min with PBS, treated with secondary antibody (1:500 anti-rabbit IgG Alexa Fluor 488 ab150077 or anti-mouse IgG Alexa Fluor 488 ab 150113, Abcam, Cambridge, MA, United States), and incubated in the dark for 2 h at room temperature. Cover-slips were washed three times for 5 min with PBS. F-actin was stained using ActinRed^TM^ 555 ReadyProbes^TM^ reagent (R37112, Invitrogen, Eugene, OR, United States) and nuclei were stained with NucBlue^TM^ Live Cell Stain ReadyProbes^TM^ reagent (R37605, Invitrogen, Eugene, OR, United States) for 30 min at room temperature. Cover-slips were washed a final time, mounted with ProLong^TM^ Glass Antifade Mountant (Invitrogen, Eugene, OR, United States) and allowed to cure for 24 h. Imaged were acquired with a FluoView 1000 Filter Confocal Microscope (Olympus, Center Valley, PA, United States). Images were median-filtered (Singh et al., 2012, 2013) and analyzed by FIJI software.

### Cell Proliferation Assay

The proliferation of hCPCs was performed using XTT reagent (Cayman Chemical, Ann Arbor, MI, United States) for cells cultured at 21, 5, and 1% O_2_. Briefly, 6,000 cells were seeded in replicate wells of 96-well plates for analysis at time 0 and after 24 h. XTT reagent was prepared per the manufacturer’s instructions, mixed with cells and media blanks, and incubated at 37°C at their respective oxygen concentrations for 2 h then read with a spectrophotometer at 450 nm for 1 s (Victor300, Perkin Elmer, Waltham, MA, United States). Absorbance values were blank-corrected and proliferation was calculated relative to their time 0 value. Data are plotted as fold increase relative to time 0, mean ± SD, *n* = 4.

### Apoptosis Flow Cytometry

Cells were cultured at 21, 5, and 1% O_2_ for 48 h then harvested for staining with the ApoDETECT AnnexinV-FITC kit (Invitrogen, Thermo Scientific, Waltham, MA, United States) to assess apoptosis without any additional stress. Cells were trypsinized, collected, and spun down 10 min at 800 *g*. The cell pellet was resuspended in 1 ml ice-cold PBS and transferred to a 1.5 ml microcentrifuge tube and spun down 1 min at 3000 rpm. The cell pellet was resuspended in 1X binding buffer and counted, volume was adjusted so cell density was 2–5 × 10^5^ cells/ml. 190 μl cell suspension was combined with 10 μl of Annexin V-FITC, mixed gently, and incubated at room temperature for 10 min. Cells were washed 1X with binding buffer, spun down 1 min at 3000 rpm, and resuspended in 190 μl binding buffer. 10 μl of 20 μg/ml propidium iodide stock solution was added to cells and incubated at room temperature for 10 min. Cells were spun down 1 min at 3000 rpm and resuspended in 200 μl of PBS. Cells were analyzed by flow cytometry using a FACS Calibur flow cytometer (Becton Dickinson, Franklin Lakes, NJ, United States), with single dye controls determining gate parameters. Apoptotic cells are positive for Annexin V-FITC and negative for PI, dead cells are dual positive, and live cells show little to no fluorescence.

### Analysis of Gene Expression

#### RNA Isolation

Human CPCs were cultured at the three distinct oxygen concentrations for 48 h. Cells were then lysed with TRIzol (Invitrogen, Carlsbad, CA, United States) for 3 min at room temp. Total RNA was then isolated using the Direct-zol RNA miniprep kit (Zymo Research, Irvine, CA, United States) per manufacturer’s instructions with on-column DNA digestion and analyzed with a spectrophotometer (NanoDrop2000, Thermo Fisher, Pleasanton, CA, United States) for quantity and purity.

#### cDNA Synthesis

200 ng of total RNA was used to prepare cDNA using the RT^2^ First Strand Kit (330404, Qiagen, Germantown, MD, United States) per manufacturer’s protocol with synthesis performed for 1 h at 37°C. Reactions were prepared from biological triplicates at the same time, with the same master mix.

#### qPCR Analysis

cDNA reactions were analyzed with RT^2^ SYBR Green ROX qPCR Mastermix (330523, Qiagen, Germantown, MD, United States) with the human hypoxia signaling pathway array (PAHS-032Z, Qiagen, Germantown, MD, United States) according to manufacturer’s instructions. Sample master mixes were thoroughly mixed and 25 μl was added to each well of the array plate using an electronic pipette. The reaction was performed on a QuantStudio 3 thermocycler and Ct values were determined by the QuantStudio Design & Analysis Software, version 1.4.1 (Thermo Fisher, Waltham, MA, United States). Relative quantification was performed using the 2^–ΔΔ*Ct*^ method (Livak and Schmittgen, 2001) with normalization to five stably expressed housekeeping genes (ATR, CTSA, HIF1AN, LGALS3, RBPJ) and fold-change calculated relative to 21% O_2_ cells. Data are shown mean ± SD, *n* = 3.

### Isolation of EVs

Human CPC-derived EVs were derived from the cell-conditioned medium collected at each oxygen concentration after culturing for 48 h in media supplemented with exosome-depleted FBS. Conditioned media was clarified with a 0.22 μm syringe filter and concentrated with an Amicon-Ultra 100 kD centricon. EVs were precipitated using ExoQuick-TC^®^(System Biosciences,PaloAlto, CA, United States) according to the manufacturer’s protocol. EVs were then suspended in PBS and stored at −80°C.

### Nanoparticle Tracking Analysis (NTA) of EVs

An equal number of cells were seeded to 10 cm dishes and EVs were isolated from equal volumes of conditioned media from normoxic and low-oxygen hCPCs. Isolated EVs were suspended in equal volumes of PBS and analyzed for size, concentration, and distribution with a NanoSight NS300 (Malvern Panalytical, Malvern, United Kingdom). Camera setting and detection threshold were kept constant for all samples for direct comparison, samples were analyzed in triplicate, and a total of at least 1000 validated tracks per sample was measured (NTA v3.3, Malvern Panalytical, Malvern, United Kingdom). Curves represent mean of triplicate measurements.

### Cryo-TEM of Isolated EVs

EVs isolated from 21, 5, and 1% O_2_ culture were sent for Cryo-TEM processing and imaging, as described in Gao et al. (2014). Briefly, a FEI Vitrobot Mark IV plunge freezer (Thermo Scientific, Waltham, MA, United States), set at room temperature and ∼95% humidity, was used to prepare vitrified cryo-TEM specimens from the aqueous samples. About 2.5 μL of the solution was applied to a TEM grid coated with lacey carbon film. After blotting using two filter papers, the grid was plunge-frozen in liquid ethane (Cavalier et al., 2009). The vitrified specimen was mounted onto a Gatan 626.DH cryo-TEM holder and transferred into a FEI Tecnai F20 TEM equipped with a Gatan twin blade retractable anti-contaminator. The cryo-TEM observation was carried out at ∼−174°C.

### Exosome Antibody Array of Isolated EVs

Isolated EVs from 21, 5, and 1% O_2_ cultured hCPCs were analyzed for expression of known exosome markers using the Exo-Check Exosome Antibody Array (System Biosciences, Palo Alto, CA, United States) per the manufacturer’s instructions. Briefly, 50 μg of EVs (by total EV protein) were added to 10X lysis buffer and vortexed. Labeling reagent was added, sample was vortexed, and incubated at room temperature with orbital shaking for 30 min. Excess labeling reagent was removed with provided columns and lysate was added to Blocking Buffer and mixed by inversion. The array membrane was incubated in distilled water at room temperature for 2 min, the water was then decanted, the lysate/blocking buffer mixture was added to the membrane, and it was incubated overnight at 4°C on a rocker. The lysate/blocking mixture was decanted, the membrane was washed at room temperature twice for 5 min with rocking. Detection Buffer was added to the membrane and incubated for 30 min at room temperature with rocking. Detection buffer was decanted and the membrane was washed three times for 5 min at room temperature. SuperSignal^TM^ West Femto Maximum Sensitivity Substrate (Thermo Scientific, Waltham, MA, United States) was used to develop the membrane by mixing reagents 1:1 and incubating on the membrane for 5 min. Imaging was performed with an Azure c600 Imaging System (Azure Biosystems, Dublin, CA, United States).

### Wound Healing (Scratch Assay) for Cell Migration

Triplicate wells of a 24-well plate were seeded with bovine aortic endothelial cells (BAECs) cultured at 37°C, 5% CO_2_, 21% O_2_, and grown to confluence. A scratch was made down the center of each well with a 200 μl standard pipet tip. Human CPC-derived EVs were added to cells at 100 μg/mL and imaged at 0 and 8 h time points. An EVOS FL Auto 2 (Thermo Scientific, Waltham, MA, United States) with a programmable stage was used to take images of the same fields over time. The wound area was analyzed for four fields per well with ImageJ by a blinded operator. The area of closure was calculated as A = (1- (*n* h area/0 h area)) x 100% per frame then averaged per well.

### Tube Formation Assay for Angiogenesis

Bovine aortic endothelial cells cultured at 37°C, 21% O_2_ were seeded in quadruplicate in Geltrex-coated wells on a 24-well plate. Human CPC-derived EVs from normoxic 21% O_2_, physoxic (5% O_2_), hypoxic (1% O_2_) conditions or an equal volume of PBS (No-EVs Control) were added to BAECs at 100 μg/mL. Plates were incubated at normoxic conditions (37°C, 5% CO_2_, 21% O_2_) for 16 h. Wells were gently washed with PBS and fixed with 4% paraformaldehyde in PBS for 20 min at room temperature. Images were taken with a 4X objective on a Leica DM IL LED microscope (Leica Microsystems, Inc., Deerfield, IL, United States). Tube formation was analyzed with the Angiogenesis Analyzer plug-in on ImageJ. Six images per well were analyzed, with four wells per group. Well totals were calculated and normalized to the No-EVs Control.

### Statistical Analysis

Data were analyzed with a one-way ANOVA and Holm-Sidak post-test, when meeting the assumptions of normality and equal variance. A *p*-value <0.05 was considered statistically significant. All values are expressed as mean ± SD.

## Results

### Cell Morphology and Cardiac Marker Expression of hCPCs

**FIGURE 1:**
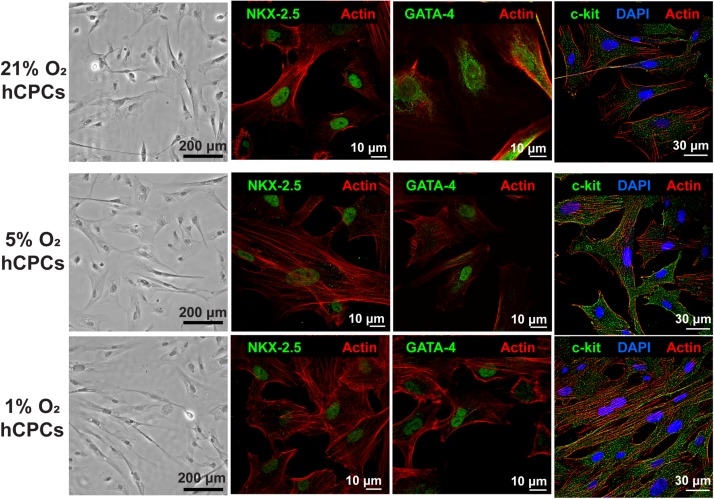
hCPCs morphology and cardiac gene expression under normoxic and hypoxic microenvironments. Human CPCs were cultured under 21, 5, and 1% O_2_ for 48 h. DIC imaging shows the typical morphology of cells, which is unchanged under hypoxia. Immunofluorescent staining for cardiac lineage markers NKX-2.5 (green, nuclear), GATA-4 (green, nuclear), and c-kit (green) showed their expression was maintained under all oxygen conditions. Nuclei are stained blue and F-actin is stained red.

Human CPCs were cultured at each oxygen concentration for 48 h with hypoxia which was achieved in a regulated hypoxia chamber at 37°C and 5% CO_2_, with humidity. Phase microscopy images were taken and revealed that hCPCs showed no changes in cell morphology compared to normoxic 21% O_2_ when subjected to low-oxygen (Figure 1). Immunofluorescent staining for cardiac markers NKX-2.5 and GATA-4 was preformed to confirm their cardiac lineage (Figure 1 and Supplementary Figures 1, 2), which confirmed that low-oxygen culture conditions did not alter their cardiac lineage. Similarly, immunostaining for c-kit was also performed and results showed that cells maintained c-kit expression under low-oxygen conditions as well (Figure 1 and Supplementary Figure 3). Therefore, low-oxygen culture did not affect CPC morphology or identity.

### Analysis of Cellular Health Under Hypoxic Culturing Conditions

**FIGURE 2:**
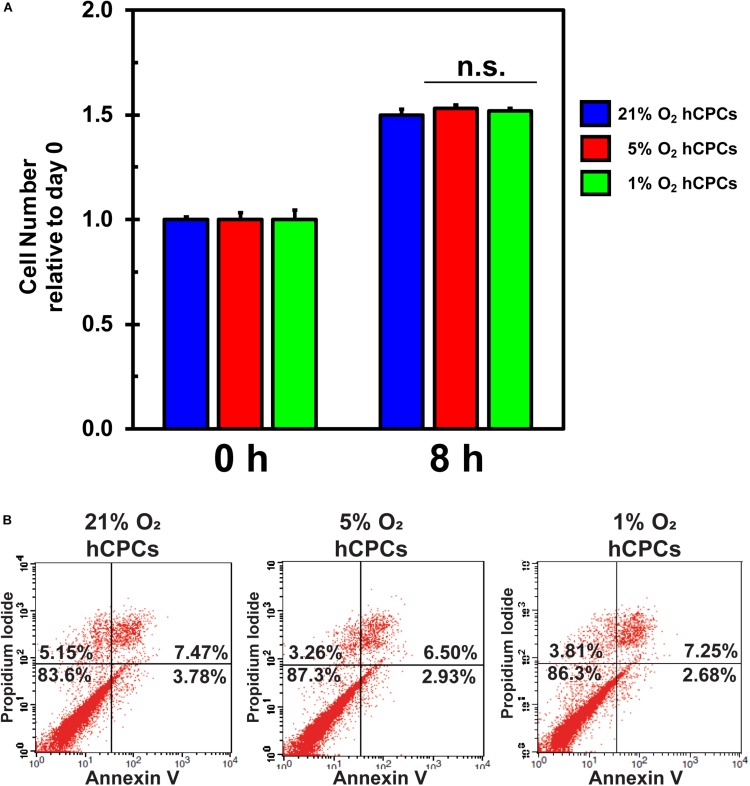
hCPCs maintain normal function under hypoxia. hCPCs were cultured under 21, 5, and 1% O_2_. **(A)** XTT assay for cell proliferation shows that cells maintained a similar level of proliferation over 24 h. Data represents mean ± SD, *n* = 4. **(B)** Flow cytometry for Annexin V/PI show that cells are equally healthy under the various oxygen conditions as the number of non-apoptotic/non-necrotic cells remains similar after 48 h of culture.

Hypoxia is known to negatively affect cellular health due to oxidative stress. To test whether 5% O_2_ and 1% O_2_ negatively affected cellular health we analyzed cell proliferation and cell death for all conditions. Cells were analyzed by XTT assay at time 0 and after 24 h. Results demonstrated that cell proliferation was unchanged under either low-oxygen condition as compared to 21% O_2_ (Figure 2A), thus the cells were dividing as normal. Furthermore, cells were analyzed for apoptosis and cell death to determine whether culturing under low-oxygen conditions was stressful and induced apoptosis or necrosis. We incubated the cells under the three oxygen concentrations for 48 h then harvested and stained the cells with Annexin V-FITC and propidium iodide (PI) for analysis by flow cytometry. Results showed that the percentage of healthy cells (dual negative) is similar for all oxygen conditions (Figure 2B). Thus, culturing cells at 5 and 1% O_2_ for 48 h did not induce apoptosis or necrosis.

### Changes in Cellular Gene Expression Under Varying O_2_ Environments

**FIGURE 3:**
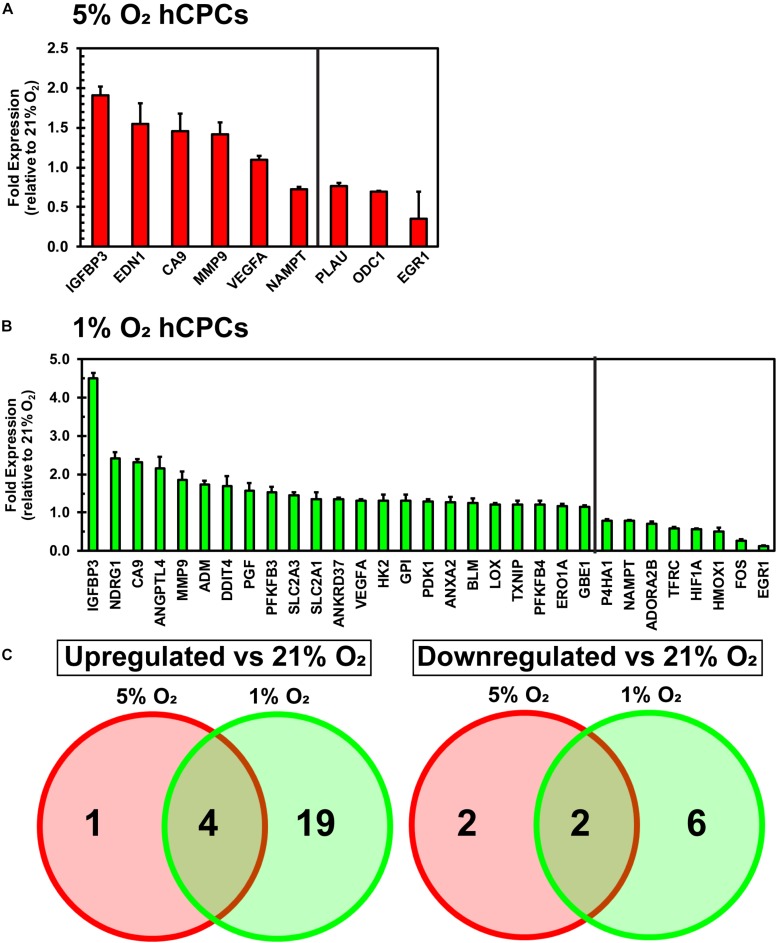
Culturing hCPCs under hypoxia modulates hypoxic gene expression. hCPCs were cultured under 21, 5, and 1% O_2_ for 48 h then total RNA was harvested and gene expression analyzed via real-time PCR. Gene expression was normalized to five housekeeping genes and calculated as a fold change relative to levels at 21% O_2_. **(A)** 5% O_2_ culturing of hCPCs significantly increased expression of five genes, and significantly decreased expression of four genes. Data represented as mean ± SD, *n* = 3, all have *p* < 0.05 as compared to 21% O_2_. **(B)** 1% O_2_ culture altered expression of numerous hypoxia-related genes. 23 genes were significantly increased in expression and 8 genes were significantly decreased in expression, as compared to 21% O_2_. Data represented as mean ± SD, *n* = 3, all have *p* < 0.05 as compared to 21% O_2_. **(C)** Venn diagram illustrating similarly and differentially regulated genes under 5 and 1% O_2,_ as compared to 21% O_2_.

A qPCR array for hypoxia-related genes was performed to assess the changes in gene expression of hCPCs under normoxic, physoxic, and hypoxic culture. Cellular gene expression was analyzed for all three oxygen conditions after 48 h of culture using an array with 84 genes involved in the hypoxia signaling pathway including: HIF1α and its co-transcription factors and other interactors; hypoxic responsive genes involved in: angiogenesis, coagulation, DNA damage and repair, metabolism, regulation of apoptosis, regulation of cell proliferation, transcription factors, transporters, channels, receptors, and others. Data were normalized to five housekeeping genes and fold change was calculated relative to the normoxic cellular expression level. Culturing under 5% O_2_ induced a significant (*p* < 0.05, *n* = 3) increase in the expression of five genes (IGFBP3, EDN1, CA9, MMP9, VEGFA) and a significant (*p* < 0.05, *n* = 3) decrease in the expression of four genes (NAMPT, PLAU, ODC1, EGR1) (Figure 3A). Culturing at 1% O_2_ caused a significant (*p* < 0.05, *n* = 3) increase in the expression of 23 genes (IGFBP3, BDRG1, CA9, ANGPLT4, MMP9, ADM, DDIT4, PGF, PFKFB3, SLC2A3, SLC2A1, ANKRD37, VEGFA, HK2, GPI, PDK1, ANXA2, BLM, LOX, TXNIP, PFKFB4, ERO1A, GBE1) and a significant (*p* < 0.05, *n* = 3) decrease in the expression of eight genes (P4HA1, NAMPT, ADORA2B, TFRC, HIF1A, HMOX1, FOS, EGR1) (Figure 3B). While the upregulated genes show some commonality, increased expression of EDN1 was unique to 5% O_2_ culture (Figure 3C). Entire array data can be found in Supplementary Table 1. These results demonstrate that culturing hCPCs under 5% O_2_ modulated few hypoxia-related genes and culturing hCPCs under 1% O_2_ modulated numerous hypoxic genes.

### Isolation and Characterization of hCPC-Derived EVs

**FIGURE 4:**
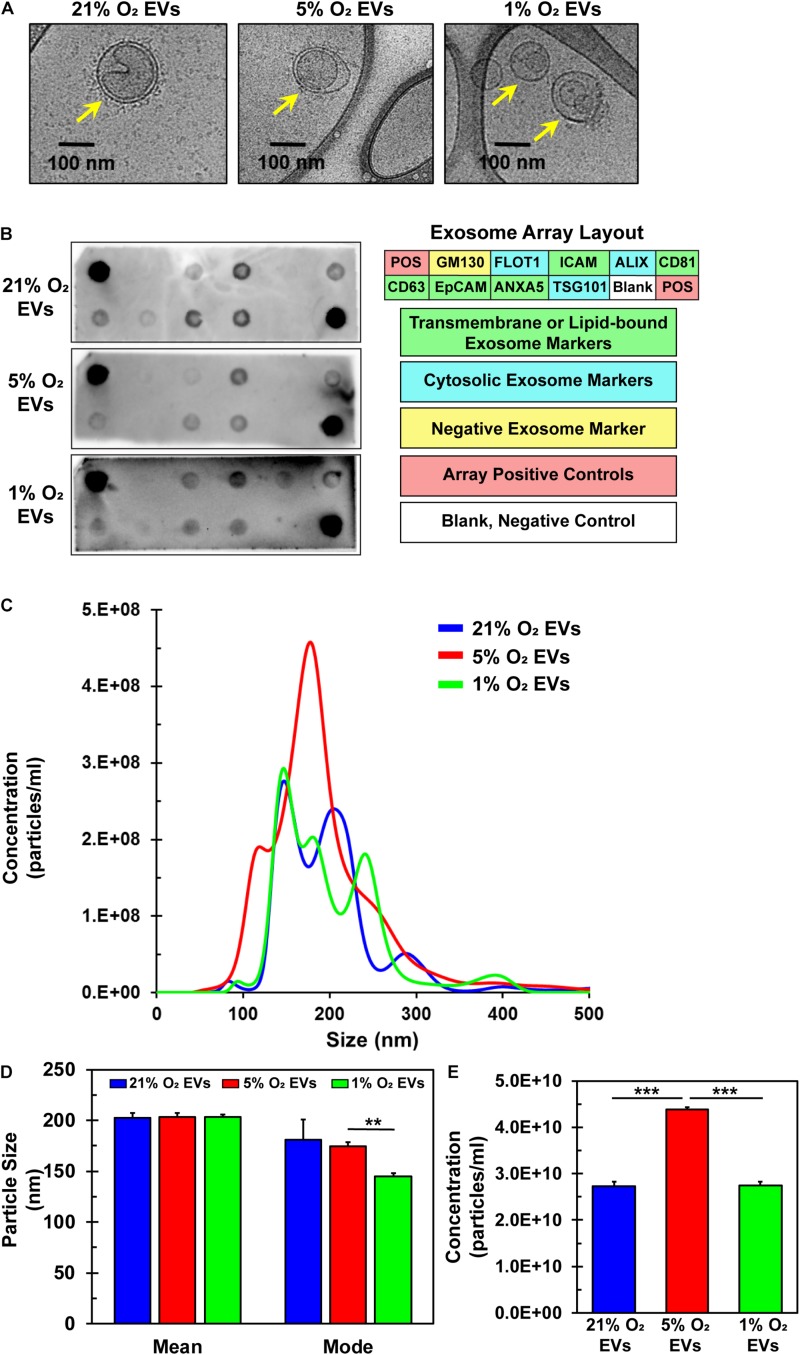
Characterization of EVs derived from hCPCs under varying oxygen microenvironment. hCPCs were cultured under three different oxygen condition (21, 5, and 1% O_2_) to generate EVs for analysis. **(A)** Cryo-TEM of isolated EVs shows their characteristic morphology of a round shape with a lipid bilayer (yellow arrows), which was identified under all three conditions. **(B)** EVs derived from 21, 5, and 1% O_2_ hCPCs were analyzed with an exosome antibody array for known exosome markers and a negative exosome marker. The isolated EVs from all three O_2_ conditions met the ISEV minimum requirements for identification. **(C)** NTA of triplicate samples shows the size and concentration distribution of EVs. **(D)** Mean and mode sizes of isolated EVs were similar and within the accepted range for extracellular vesicles, data is mean ± SD, *n* = 3. **(E)** The concentration of EVs isolated from hCPCs cultured under 5% O_2_ microenvironment was higher than 21 and 1% O_2_ groups, data is mean ± SD, *n* = 3. **p < 0.01, ***p < 0.001.

EVs were successfully isolated from hCPCs under all three O_2_ conditions and analyzed via Cryo-TEM to preserve their shape and structure. The lipid bilayer is easily identified in representative TEM images (Figure 4A, yellow arrows). EVs were further analyzed for common exosomal markers with an antibody array. To verify exosome identity, isolated particles were tested to comply with international standards (Thery et al., 2018) demonstrating at least three positive markers and one negative marker. Results show that these EVs expressed the exosomal transmembrane or lipid-bound markers ICAM, CD81, CD63, ANXA5, and cytosolic markers ALIX, FLOT-1 and TSG101 (Figure 4B) (de Gassart et al., 2003; Lotvall et al., 2014). Detection of the negative marker GM130 (Lotvall et al., 2014; Keerthikumar et al., 2015; Samaeekia et al., 2018), a Golgi-associated protein, was not seen, thus confirming their identity as exosomes. Detection patterns were similar for the EVs isolated under all three oxygen conditions. Nanoparticle tracking analysis (NTA) revealed the size and concentration distribution for EVs derived from the three oxygen conditions (Figure 4C). Their sizes are consistent with those of exosomes (50–150 nm) (Yanez-Mo et al., 2015) and small microvesicles (100–1000 nm) (Cocucci and Meldolesi, 2015). The mean and mode sizes of the particles isolated from the differing oxygen concentrations were similar (Figure 4D). Interestingly, concentration, and thus yield, of EVs was 1.6-fold greater under 5% O_2_ conditions (Figure 4E). Since EVs were isolated from identically seeded plates with equal volumes of media and were resuspended in identical volumes of PBS the concentration of EVs is congruent to their yield. Furthermore, this data showed that 5% O_2_ EVs had a unique size distribution. Collectively, these data verify the identity of the isolated particles to include small microvesicles and exosomes. Varying oxygen concentrations generated EVs that were similar in mean and mode size, however, their secretion was increased under 5% O_2_ and also had a unique size distribution pattern as compared to 21 and 1% O_2_ culture.

### Angiogenic Potential of hCPC-Derived EVs

**FIGURE 5:**
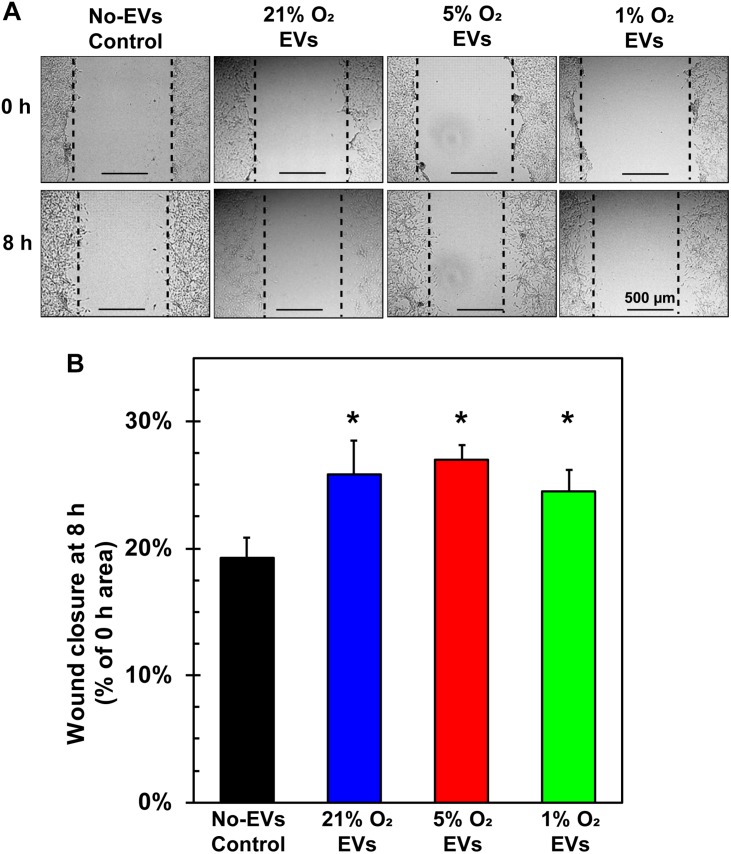
Effect of hCPC-derived EVs on endothelial cell migration. BAECs were grown to confluence, wounded with a pipet tip, and treated with 100 μg/ml of total EV protein from each condition. **(A)** Representative images from 0 and 8 h show the extent of cell migration, with dotted lines approximating the cell front. **(B)** Wound healing assay demonstrates that treating ECs with hCPC-derived EVs increased cell migration significantly when compared to No-EVs group. However, no significant differences were observed between 5 and 1% O_2_ derived EVs. Data are shown as mean ± SD, *n* = 4, *p < 0.05 vs. No-EVs control.

A key tenet of angiogenesis is cell migration as cells must migrate toward the formation site of the new vessel (Adair and Montani, 2010). The wound-healing/scratch assay is a well-established assay for 2-D cell migration (Rodriguez et al., 2005). BAECs were grown to confluence in 24-well plates, wounded with a pipet tip, and treated with hCPC EVs from 21, 5, and 1% O_2_ culture at 100 μg/ml EVs (total EV protein) at time 0. Results demonstrate that all groups of EVs significantly increased BAEC migration after 8 h as compared to No-EVs control (Figure 5A). The 5% O_2_-derived EVs had the greatest percentage wound closure, however, this was not statistically significant compared to 21 or 1% O_2_ (Figure 5B). These results indicate that hCPC-derived EVs increase endothelial cell migration, a crucial component of angiogenesis, but this was not enriched by generation of EVs under hypoxic conditions.

**FIGURE 6:**
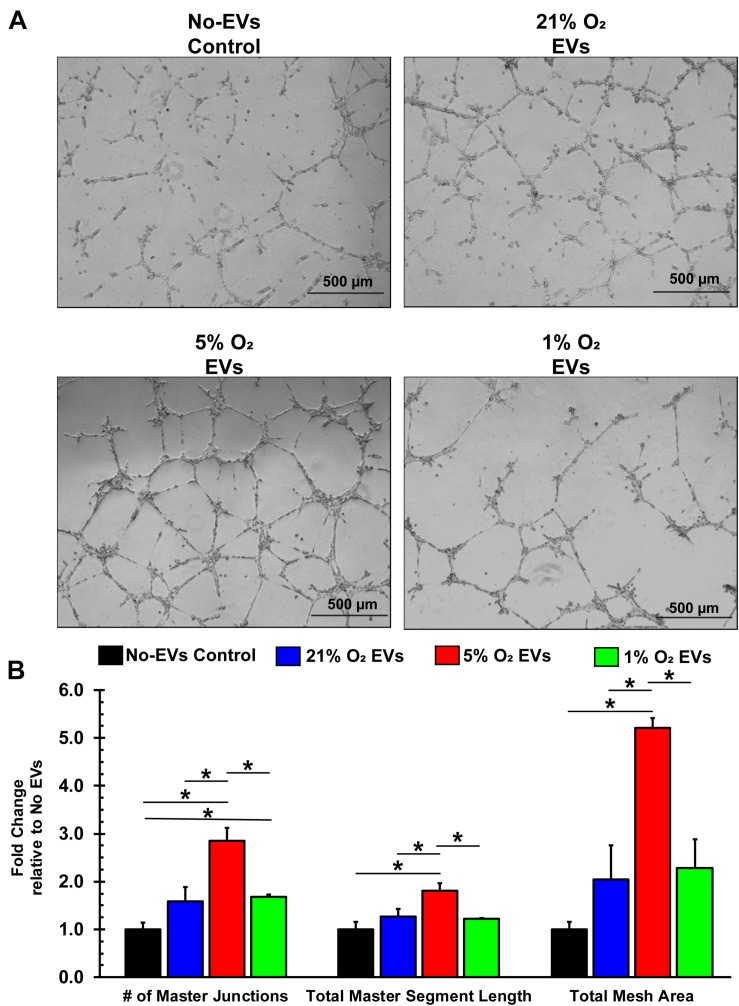
EVs derived from hCPCs under physoxia enhances angiogenesis *in vitro*. BAECs were seeded onto Matrigel-coated wells with 100 μg/ml of total EV protein from each condition and incubated for 16 h. **(A)** Representative images illustrate the degree of tube formation for each group. **(B)** 1% O_2_-derived EVs significantly increased tube formation in terms of the number of master junctions as compared to No-EVs control. However, 5% O_2_-derived EVs significantly increased tube formation as compared to all groups in terms of the number of master junctions, the total master segment length, and the total mesh area. Data shown represents mean ± SD, *n* = 3, *p < 0.05 for comparison.

Additionally, endothelial cells that are to form new blood vessels must reorganize into 3D tubules to allow for subsequent blood flow (Adair and Montani, 2010). The tube formation assay using ECs is a well-characterized model system for *in vitro* angiogenesis (Arnaoutova et al., 2009), which we used to assess the hCPC-derived EVs. BAECs were seeded with media containing 100 μg/ml EVs (total EV protein) from 21, 5, and 1% O_2_ cultured hCPCs onto basement membrane-coated wells and allowed to form tubules for 16 h (Figure 6A). Results demonstrate that treatment with 5% O_2_-derived EVs has shown the greatest enhancement of tube formation. These EVs significantly (*p* < 0.05, *n* = 3) increased the number of master junctions, total master segment length, and total mesh area as compared to No-EV control (Figure 6B). Impressively, 5% O_2_ hCPC-derived EVs also significantly (*p* < 0.05, *n* = 3) enhanced tube formation by all measurements as compared to both 21 and 1% O_2_ EV treatment (Figure 6B). Thus, culturing hCPCs at 5% O_2_ generated EVs with the greatest angiogenic potential in terms of tube formation.

## Discussion

Overall, this study demonstrated the therapeutic potential of human CPC-derived EVs for cardiac repair. Our study elucidated the optimal oxygen concentration required for culturing hCPCs and showed that the EV-cargo released under 5% O_2_ is very potent for promoting angiogenesis. Furthermore, only a few hypoxic genes were modulated under 5% O_2_ conditions, when compared to numerous genes altered under 1% O_2_ culturing. Our results clearly demonstrate that physoxic (5% O_2_) culturing of hCPCs may play a crucial role in myocardial repair applications, especially during cell transplantation studies to prevent hypoxic shock for transplanted cells in the ischemic heart. Furthermore, physoxic culturing also influences the EV-cargo released by hCPCs as a source for cell-free therapeutics.

In this study, we have analyzed hCPCs and the functionality of their derived exosomes cultured under three different micro-environments (21, 5, and 1% O_2_). Our results denote that hCPCs cultured under a low-oxygen, physiological microenvironment (a.k.a physoxic, 5% O_2_) were able to maintain normal cell morphology and expression of cardiac markers, thereby demonstrating their robustness under physoxic oxygen conditions. NTA analysis indicated our ability to successfully isolate exosomes from cells under all oxygen conditions. Cells cultured at 5% O_2_ exhibited increased exosome secretion with a unique size and concentration profile as compared to normoxia and 1% O_2_ culturing. Functional assays from this study signify our novel hypothesis that physoxic oxygen concentration affects the therapeutic potential of hCPC-derived EVs. Additionally, gene expression profiling of the hCPCs revealed a possible molecular mechanism underlying the increased potency of 5% O_2_-derived EVs. On the other hand, EDN1 was uniquely and significantly upregulated in 5% O_2_ cultured hCPCs when compared to normoxia or 1% O_2_ cultured hCPCs. *EDN1* encodes endothelin 1 (ET-1), a secreted peptide that acts as a paracrine signaling factor mediating growth (Soh et al., 2016), survival (Del Bufalo et al., 2002; Nelson et al., 2005), and angiogenesis (Salani et al., 2000; Wulfing et al., 2004; Wu et al., 2014). Future proteomic analysis of exosomes derived from 5% O_2_ cultured hCPCs would reveal if EDN1 is delivered to target cells via exosomes and play a crucial role promoting angiogenesis.

Our NTA analysis results showed an increased EV release under 5% O_2_ condition, but not under at 1% O_2_. Other studies have observed increased secretion of EVs under hypoxia (King et al., 2012; Bian et al., 2014; Zhu J. et al., 2018; Patton et al., 2019; Zhang et al., 2019), however these studies were not performed with hCPCs. Multiple studies have demonstrated enhanced functional effects of hypoxia-derived versus normoxia-derived EVs. A study of MSC-EVs observed increased proliferation, migration, and tube formation with hypoxia-derived EVs (1% O_2_ for 72 h) as compared to those from normoxic conditions (Bian et al., 2014). Another study on glioma cells observed significantly increased tube formation by endothelial progenitor cells when treated with hypoxia (<0.5% O_2_ for undisclosed time) versus normoxia-derived EVs; however, the treatments equally protected glioma cells from oxidative stress (Kore et al., 2018). Similarly, human cardiosphere-derived EVs isolated under 18 and 1% O_2_ showed increased tube formation as compared to PBS control. However, the two conditions were either not analyzed to each other or their difference was not statistically significant (Namazi et al., 2018). Zhu L.P. et al. (2018), showed that intramyocardial implantation of EVs generated from BM-MSCs under hypoxia (1% O_2_ for 72 h) significantly decreased scar formation and improved cardiac function 28 days post-MI, as compared to normoxia-derived EVs. Overall, the results from our findings highlight the therapeutic potential of physoxia generated EVs as compared to normoxia-derived EVs. Wound healing assays showed that EV treatment significantly increased migration as compared to No-EVs control. Similarly, 5% O_2_ hCPC-derived exosomes showed significant increase in tube formation as assessed by the number of master junctions, segment length, and mesh area as compared to both 21 or 1% O_2_ EVs. Ultimately, 5% O_2_ CPC-derived EVs in this study displayed the greatest therapeutic effect *in vitro* as a result of enhanced angiogenic behavior.

## Conclusion

Promising results have stemmed from our study by assessing the therapeutic potential of hCPC-derived EVs under physoxia. Culturing hCPCs under physoxia showed increased EVs secretion and minimal changes in cellular expression of hypoxia-related genes. Future studies will focus on functional outcome of hCPC-derived EVs for myocardial repair *in vivo* and to perform proteomics and miRNA profiling of EV cargo to identify novel proteins and miRNAs modulated under physoxia.

## Data Availability Statement

The datasets generated for this study are available on request to the corresponding author.

## Author Contributions

MK, NP, and JD conceived and designed the experiments. MK, NP, JD, NK, SR, and HS performed the experiments and analyzed the data. MK, CC, HS, and MA contributed reagents, materials, and analysis tools. JD, NP, and MK wrote the first draft of the manuscript. All authors contributed to manuscript revision, read and approved the submitted version.

## Conflict of Interest

The authors declare that the research was conducted in the absence of any commercial or financial relationships that could be construed as a potential conflict of interest.
