# Supplementary material for: Human Cardiac Progenitor Cells Enhance Exosome Release and Promote Angiogenesis Under Physoxia
Source: Front Cell Dev Biol. 2020 Mar 6;8:130. doi: 10.3389/fcell.2020.00130 (PMC7068154; doi:10.3389/fcell.2020.00130)
Supplement: Supplementary file 1 [file Data_Sheet_1.PDF]

Supplementary Figure 1

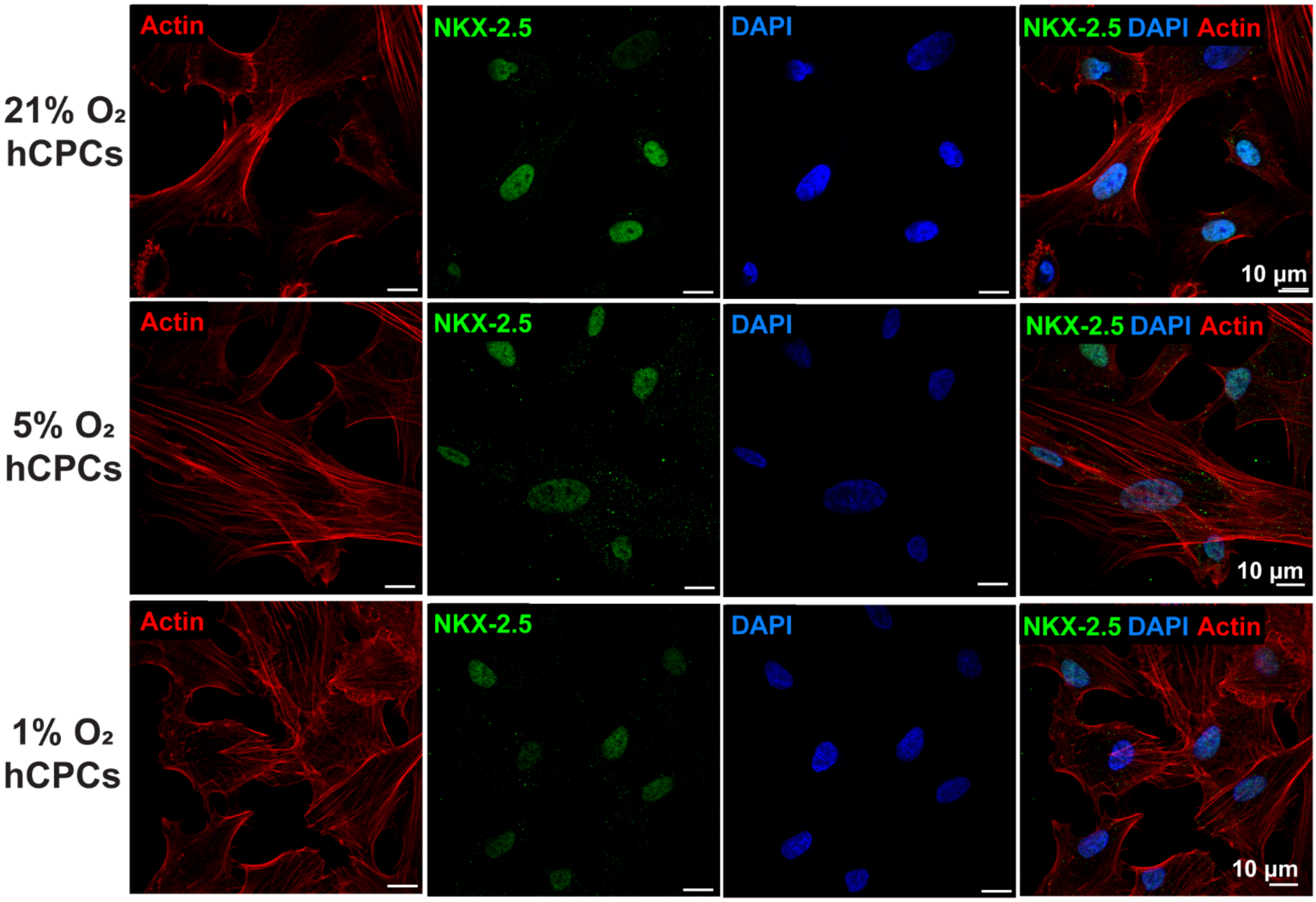

Supplementary Figure 1. NKX-2.5 expression under low-oxygen culture. hCPCs were cultured for 48 h under 21%, 5%, and 1% O2 then fixed and stained for the cardiac transcription factor NKX2.5 (green). NucBlue stain was used to label the nucleus (blue) and ActinRed 555 stain labeled F-actin (red). Nuclear expression of this cardiac lineage marker was maintained under low-oxygen culture. Scale bar is 10 µm.

Supplementary Figure 2

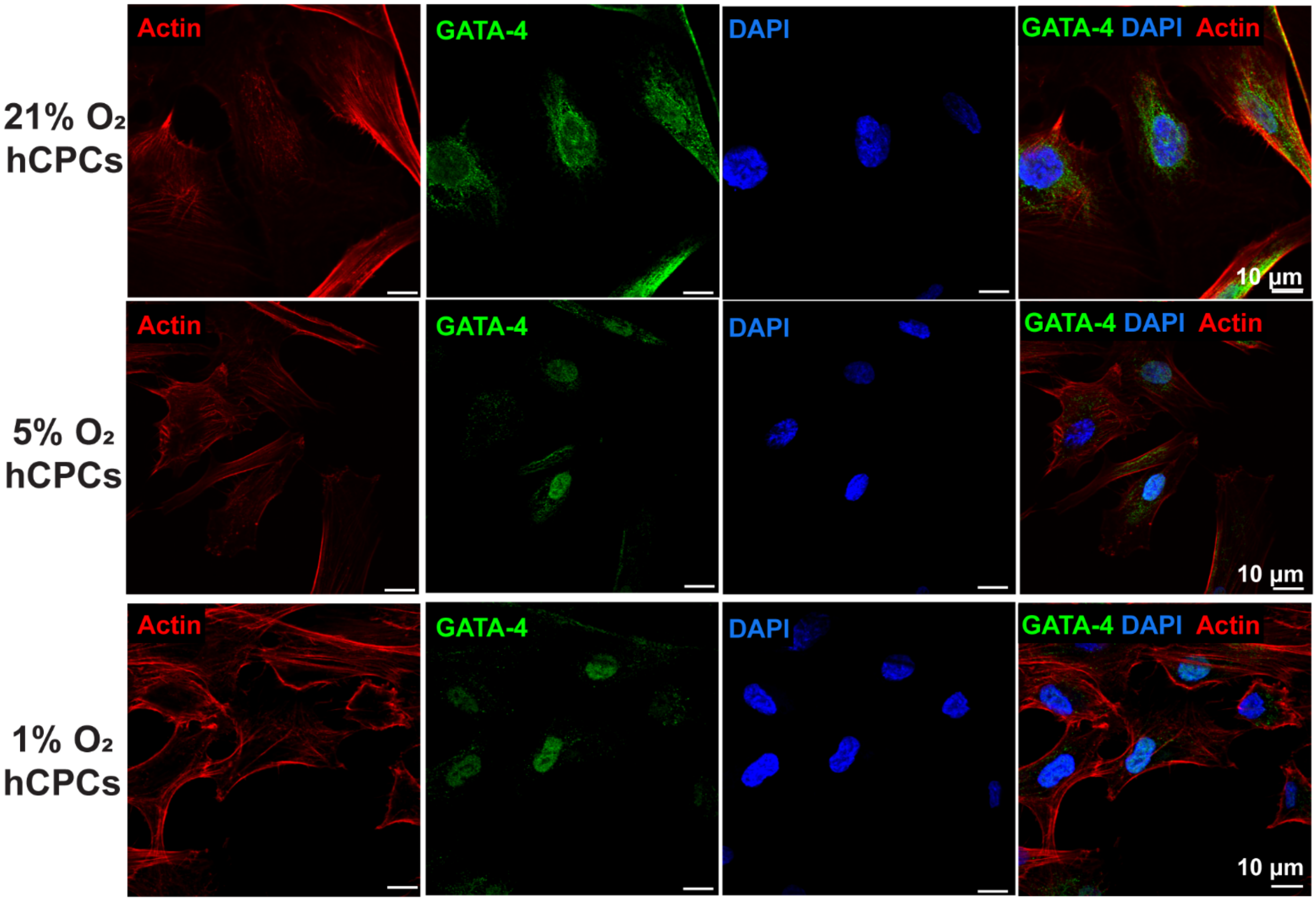

Supplementary Figure 2. GATA-4 expression under low-oxygen culture. hCPCs were cultured for 48 h under 21%, 5%, and 1% O2 then fixed and stained for the cardiac transcription factor GATA-4 (green). NucBlue stain was used to label the nucleus (blue) and ActinRed 555 stain labeled F-actin (red). Nuclear expression of this cardiac lineage marker was maintained under low-oxygen culture. Scale bar is 10 μm.

Supplementary Figure 3

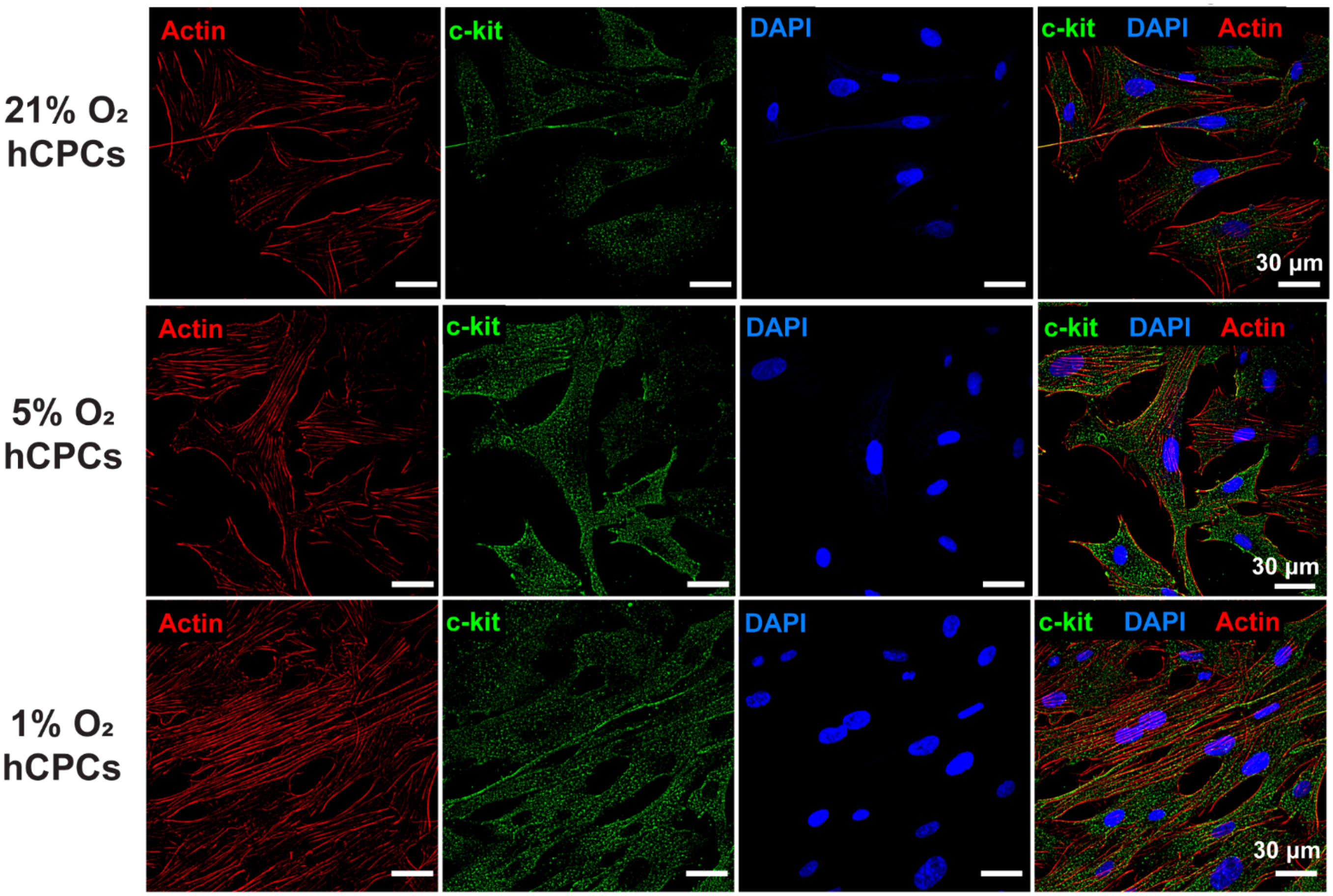

Supplementary Figure 3. c-kit expression under low-oxygen culture. hCPCs were cultured for 48 h under 21%, 5%, and 1% O2 then fixed and stained for cellular receptor c-kit(green). NucBlue stain was used to label the nucleus (blue) and ActinRed 555 stain labeled F-actin (red). Robust expression of this cell marker was maintained under low oxygen culture. Scale bar is 30 μm.

Supplemental Table 1. Summary of qPCR data analysis performed on CPCs cultured under 21%, 5%, and 1% O_2.

		Fold expression relative to 21% O_2, n=3							
		21% O2		5% O2			1% O2		
Symbol	Gene	Fold Exp	SD	Fold Exp	SD	p vs 21	Fold Exp	SD	p vs 21
ADM	Adrenomedullin	1.002	0.083	1.171	0.098	0.0849	1.737	0.101	0.0006
ADORA2B	Adenosine A2b receptor	1.003	0.098	0.871	0.095	0.1692	0.701	0.057	0.0099
ALDOA	Aldolase A, fructose-bisphosphate	1.003	0.100	1.065	0.101	0.4920	1.196	0.116	0.1449
ANGPTL4	Angiopoietin-like 4	1.002	0.074	1.099	0.211	0.4942	2.147	0.303	0.0031
ANKRD37	Ankyrin repeat domain 37	1.002	0.085	0.964	0.197	0.7743	1.349	0.044	0.0033
ANXA2	Annexin A2	1.001	0.043	0.938	0.014	0.0733	1.261	0.151	0.0455
APEX1	APEX nuclease (multifunctional DNA repair enzyme) 1	1.008	0.153	0.926	0.363	0.7367	1.014	0.171	0.9660
ARNT	Aryl hydrocarbon receptor nuclear translocator	1.043	0.389	1.039	0.227	0.9885	1.059	0.137	0.9497
BHLHE40	Basic helix-loop-helix family, member e40	1.000	0.026	1.045	0.090	0.4522	1.139	0.120	0.1214
BLM	Bloom syndrome, RecQ helicase-like	1.000	0.019	0.942	0.059	0.1804	1.250	0.128	0.0287
BNIP3	BCL2/adenovirus E1B 19kDa interacting protein 3	1.001	0.040	0.899	0.160	0.3444	0.978	0.045	0.5444
BNIP3L	BCL2/adenovirus E1B 19kDa interacting protein 3-like	1.005	0.122	0.915	0.267	0.6235	1.220	0.116	0.1449
BTG1	B-cell translocation gene 1, anti-proliferative	1.006	0.132	0.778	0.261	0.2483	1.144	0.112	0.2395
CA9	Carbonic anhydrase IX	1.005	0.119	1.460	0.216	0.0330	2.317	0.086	0.0001
CCNG2	Cyclin G2	1.011	0.192	0.848	0.240	0.4103	1.036	0.115	0.8560
COPS5	COP9 constitutive photomorphogenic homolog subunit 5 (Arabidopsis)	1.009	0.157	0.955	0.333	0.8120	0.998	0.068	0.9167
DDIT4	DNA-damage-inducible transcript 4	1.000	0.020	1.128	0.245	0.4181	1.699	0.263	0.0101
DNAJC5	DnaJ (Hsp40) homolog, subfamily C, member 5	1.001	0.064	0.860	0.077	0.0713	0.949	0.109	0.5155
EDN1	Endothelin 1	1.006	0.140	1.549	0.258	0.0328	0.741	0.235	0.1687
EGLN1	Egl nine homolog 1 (C. elegans)	1.001	0.063	0.976	0.220	0.8591	1.106	0.120	0.2508
EGLN2	Egl nine homolog 2 (C. elegans)	1.003	0.089	0.940	0.128	0.5225	1.114	0.209	0.4450
EGR1	Early growth response 1	1.006	0.138	0.358	0.340	0.0377	0.133	0.018	0.0004
EIF4EBP1	Eukaryotic translation initiation factor 4E binding protein 1	1.004	0.113	0.962	0.291	0.8272	1.169	0.162	0.2215
ENO1	Enolase 1, (alpha)	1.006	0.127	0.937	0.323	0.7479	1.346	0.185	0.0585
ERO1A	ERO1-like (S. cerevisiae)	1.002	0.076	0.939	0.172	0.5928	1.170	0.052	0.0342
F10	Coagulation factor X	1.005	0.117	1.240	0.348	0.3560	0.957	0.101	0.6192
F3	Coagulation factor III (thromboplastin, tissue factor)	1.029	0.310	0.821	0.456	0.5492	1.220	0.324	0.5016
FOS	FBJ murine osteosarcoma viral oncogene homolog	1.001	0.049	0.999	0.116	0.9794	0.273	0.035	<0.0001
GBE1	Glucan (1,4-alpha-), branching enzyme 1	1.001	0.064	0.990	0.125	0.8986	1.141	0.041	0.0332
GPI	Glucose-6-phosphate isomerase	1.002	0.075	1.063	0.104	0.4562	1.301	0.170	0.0495
GYS1	Glycogen synthase 1 (muscle)	1.007	0.144	0.812	0.207	0.2515	1.199	0.608	0.6228
HIF1A	Hypoxia inducible factor 1, alpha subunit (basic helix-loop-helix transcription factor)	1.002	0.067	0.678	0.080	0.0058	0.569	0.011	0.0004
HIF3A	Hypoxia inducible factor 3, alpha subunit	1.003	0.099	0.756	0.150	0.0760	1.273	0.168	0.0745
HK2	Hexokinase 2	1.001	0.042	0.895	0.135	0.2639	1.311	0.158	0.0304
HMOX1	Heme oxygenase (decycling) 1	1.002	0.078	0.830	0.125	0.1132	0.501	0.113	0.0032
HNF4A	Hepatocyte nuclear factor 4, alpha	1.010	0.167	0.791	0.859	0.6870	1.358	0.338	0.1851
IER3	Immediate early response 3	1.009	0.169	0.709	0.105	0.0593	0.843	0.118	0.2355
IGFBP3	Insulin-like growth factor binding protein 3	1.005	0.116	1.903	0.112	0.0006	4.513	0.141	<0.0001
JMJD6	Jumonji domain containing 6	1.001	0.058	0.918	0.278	0.6393	1.051	0.153	0.6246
LDHA	Lactate dehydrogenase A	1.005	0.118	0.889	0.208	0.4481	1.129	0.061	0.1812
LOX	Lysyl oxidase	1.001	0.057	1.040	0.283	0.8265	1.220	0.038	0.0052
MAP3K1	Mitogen-activated protein kinase kinase kinase 1	1.002	0.067	1.081	0.158	0.4699	0.902	0.107	0.2420
MET	Met proto-oncogene (hepatocyte growth factor receptor)	1.022	0.255	0.817	0.354	0.4614	1.239	0.263	0.3629
MIF	Macrophage migration inhibitory factor (glycosylation-inhibiting factor)	1.001	0.043	1.056	0.040	0.1801	1.120	0.096	0.1216
MMP9	Matrix metallopeptidase 9 (gelatinase B, 92kDa gelatinase, 92kDa type IV collagenase)	1.011	0.185	1.416	0.152	0.0428	1.848	0.219	0.0072
MXI1	MAX interactor 1	1.004	0.111	0.863	0.232	0.3961	1.209	0.077	0.0583
NAMPT	Nicotinamide phosphoribosyltransferase	1.000	0.029	0.727	0.027	0.0003	0.779	0.030	0.0008
NCOA1	Nuclear receptor coactivator 1	1.001	0.054	0.948	0.110	0.4954	0.971	0.082	0.6247
NDRG1	N-myc downstream regulated 1	1.010	0.167	1.109	0.392	0.7079	2.424	0.161	0.0005
NFKB1	Nuclear factor of kappa light polypeptide gene enhancer in B-cells 1	1.005	0.121	0.977	0.047	0.7277	0.985	0.095	0.8329
ODC1	Ornithine decarboxylase 1	1.003	0.099	0.691	0.016	0.0057	0.845	0.184	0.2604
P4HA1	Prolyl 4-hydroxylase, alpha polypeptide I	1.000	0.029	1.085	0.152	0.3953	0.783	0.035	0.0012
P4HB	Prolyl 4-hydroxylase, beta polypeptide	1.009	0.163	1.012	0.295	0.9884	1.297	0.152	0.0888
PDK1	Pyruvate dehydrogenase kinase, isozyme 1	1.003	0.090	0.877	0.146	0.2722	1.295	0.049	0.0078
PER1	Period homolog 1 (Drosophila)	1.036	0.312	0.748	0.164	0.2299	1.311	0.381	0.3882
PFKFB3	6-phosphofructo-2-kinase/fructose-2,6-biphosphatase 3	1.000	0.029	0.885	0.127	0.2010	1.537	0.139	0.0028
PFKFB4	6-phosphofructo-2-kinase/fructose-2,6-biphosphatase 4	1.001	0.067	1.074	0.144	0.4705	1.207	0.094	0.0365
PFKL	Phosphofructokinase, liver	1.000	0.016	0.980	0.106	0.7628	1.082	0.107	0.2595
PFKP	Phosphofructokinase, platelet	1.000	0.027	0.969	0.027	0.2324	1.023	0.050	0.5219
PGAM1	Phosphoglycerate mutase 1 (brain)	1.002	0.069	0.921	0.255	0.6235	1.170	0.103	0.0788
PGF	Placental growth factor	1.000	0.038	1.030	0.105	0.6659	1.563	0.208	0.0099
PGK1	Phosphoglycerate kinase 1	1.006	0.129	0.937	0.377	0.7792	1.260	0.160	0.0990
PIM1	Pim-1 oncogene	1.003	0.097	1.152	0.154	0.2292	1.089	0.065	0.2711
PKM	Pyruvate kinase, muscle	1.001	0.055	1.028	0.154	0.7891	1.173	0.169	0.1690
PLAU	Plasminogen activator, urokinase	1.000	0.030	0.770	0.036	0.0011	1.085	0.145	0.3764
RUVBL2	RuvB-like 2 (E. coli)	1.002	0.072	0.945	0.106	0.4840	1.020	0.122	0.8366
SERPINE1	Serpin peptidase inhibitor, clade E (nexin, plasminogen activator inhibitor type 1), member 1	1.001	0.064	0.935	0.250	0.6807	1.131	0.115	0.1623
SLC16A3	Solute carrier family 16, member 3 (monocarboxylic acid transporter 4)	1.001	0.064	0.839	0.174	0.2047	1.068	0.299	0.7236
SLC2A1	Solute carrier family 2 (facilitated glucose transporter), member 1	1.000	0.023	0.982	0.116	0.8051	1.359	0.167	0.0210
SLC2A3	Solute carrier family 2 (facilitated glucose transporter), member 3	1.001	0.058	1.096	0.081	0.1739	1.445	0.089	0.0019
TFRC	Transferrin receptor (p90, CD71)	1.002	0.069	0.765	0.154	0.0718	0.578	0.058	0.0012
TP53	Tumor protein p53	1.001	0.062	1.021	0.113	0.8014	0.915	0.071	0.1892
TPI1	Triosephosphate isomerase 1	1.003	0.094	0.962	0.075	0.5866	1.028	0.115	0.7851
TXNIP	Thioredoxin interacting protein	1.002	0.076	0.850	0.223	0.3264	1.218	0.096	0.0378
USF2	Upstream transcription factor 2, c-fos interacting	1.003	0.088	0.931	0.188	0.5804	0.984	0.109	0.8258
VDAC1	Voltage-dependent anion channel 1	1.021	0.250	0.937	0.462	0.7955	1.047	0.175	0.8898
VEGFA	Vascular endothelial growth factor A	1.000	0.012	1.093	0.050	0.0351	1.318	0.038	0.0002
